# Self-reported carbohydrate supercompensation and supplementation strategies adopted by Olympic triathlon athletes

**DOI:** 10.1590/1414-431X2024e14189

**Published:** 2025-02-03

**Authors:** M.P. Mendes, A.H. Marinho, F.A. Moura, G.S. Bádue, G.A. Ferreira, G.G. de Araujo, A.E. Lima-Silva, T. Ataide-Silva

**Affiliations:** 1 Programa de Pós-graduação em Nutrição, Faculdade de Nutrição, Universidade Federal de Alagoas, Maceió, AL, Brasil; 2 Laboratório de Ciências do Esporte Aplicadas, Instituto de Educação Física e Esporte, Universidade Federal de Alagoas, Maceió, AL, Brasil; 3 Escola Superior de Educação Física, Universidade de Pernambuco, Recife, PE, Brasil; 4 Grupo de Pesquisa de Desempenho Humano, Universidade Tecnológica Federal do Paraná, Curitiba, PR, Brasil

**Keywords:** Diet, Carbohydrate intake, Glycogen, Triathlon

## Abstract

The aim of the present study was to describe the use of tapering, carbohydrate (CHO) supercompensation, and supplementation strategies self-reported by athletes in the Olympic triathlon category. A total of 72 triathletes (61 males and 11 females) answered an online questionnaire about their training and performance, supercompensation strategies, carbohydrate supplementation, and use of supplements and other ergogenic substances. The information was summarized and subjected to descriptive analysis. Shapiro-Wilk test was applied to check data normality. The *t*-test was used to investigate differences in the analyzed variables between sexes. Almost all triathletes reported to have performed tapering (93.05%) and approximately half of them adopted a CHO supercompensation strategy (48.61%); updated CHO supercompensation was the most used strategy (27.77%). Most participants (86.11%) used CHO supplementation during competitions, but in amounts below the 60 g/h recommended for most athletes (96.77%). Thus, since few triathletes performed supercompensation, in addition to the insufficient amount of supplemented carbohydrate taken by them, it could be concluded that triathletes were not sufficiently aware of nutritional recommendations or did not adopt them.

## Introduction

The preparation of athletes for endurance competitions (such as triathlon) encompasses both systematizing and integrating nutritional, training, and recovery aspects (1). With respect to nutrition, carbohydrate (CHO) supercompensation has played an essential role in optimizing the athletes' endurance performance (2). In sports nutrition, CHO supercompensation (also known as CHO loading) may be defined as the period in which the athlete intakes large amounts of CHO, which can vary from 8 to 12 g/kg, 3 to 7 days before competitions to increase glycogen stores (3). CHO supercompensation occurs before the competition, together with the tapering phase of training. The tapering phase is the moment of training in which the athlete reduces training volume and maintains intensity in order to recover physiologically and psychologically from the stress of daily training (4). The combination of increased CHO consumption and reduced training volume enables the effective increase of muscle glycogen stores before the competition (3).

Three different CHO supercompensation models were herein proposed (5-7). The “classical” CHO supercompensation model requires 3 to 4 days of intense training and low (<2 g/kg) CHO intake (the so-called muscle glycogen depletion phase), followed by 3-4 days of reduced training volume and high (<8-12 g/kg) CHO intake (the so-called muscle glycogen supercompensation phase) (5). From this model, Sherman et al. (7) developed a “modified” muscle glycogen supercompensation, which, unlike the classical model, could be done without the muscle glycogen depletion phase and thus implementing CHO supercompensation only with the muscle glycogen supercompensation phase. This “modified” CHO supercompensation model has the advantage of being more practical and avoiding potential negative effects arising from the “classical” CHO supercompensation model such as fatigue due extreme CHO restriction (<2 g/kg) (3) and strenuous training for muscle glycogen depletion (7). Another possible negative effect of the “classical” CHO supercompensation model is that it does not have a tapering phase of training, which increases the risk of injury (7). Furthermore, an “updated” CHO supercompensation model preconizes muscle glycogen supercompensation by resting and consuming 10 g/kg/day of CHO 24 h prior to competitions (6). Therefore, endurance athletes are advised to prioritize the modified or updated model, as it might be less exhausting and closer to the tapering protocol.

In addition to CHO supercompensation, CHO supplementation during competition is also a widely recommended nutritional strategy to help improve athletes' endurance performance (8). Athletes competing in endurance sports for longer than 60 min can benefit from periodic CHO intake during races (9). Plasma glucose maintenance is likely the primary mechanism explaining athletes' gain in endurance performance after CHO intake, since it leads to higher CHO oxidation rate in active muscles (10). The rate-limiting step for exogenous CHO oxidation appears to be the activity performed by sodium-dependent glucose transporter 1 (SGLT1) and GLUT-5 in the gastrointestinal tract (11). SGLT1 transports glucose, and its transport capacity appears to be limited to 1 g of glucose per min (60 g per hour), whereas GLUT-5 transports fructose at maximum transport capacity of 0.5 g fructose per min (30 g per hour) (3,12). Since competitions lasting from 1 to 2 h do not demand large amounts of exogenous CHO, the supplementation of ∼30-60 g of glucose per hour during the exercise is recommended (8). However, athletes engaging in competitions longer than 2 h may benefit from the glucose (60 g per hour)/fructose (30 g per hour) combination, since it can increase CHO oxidation rates by up to 75%, compared to exclusive intake of 60 g of glucose per hour (13).

Because CHO manipulation enhances athletes' endurance performance (2,8), CHO supercompensation prior to and supplementation during competitions are useful for triathletes. CHO supercompensation and supplementation are specifically useful in Olympic triathlon competitions, where athletes perform 1.5 km of swimming, 40 km of cycling, and 10 km of running. Elite athletes complete the entire race within approximately 110 min (14), whereas regional level athletes do it in approximately 150 min (15). Therefore, triathlons lasting longer than 90 min are categorized as endurance sports that may benefit from supercompensation strategies and CHO supplementation to improve performance (9). However, despite previous evidence showing that elite Olympic triathletes did not adequately follow CHO supplementation recommendations on competition day (16), it is still unknown whether triathletes are fully aware of and appropriately utilize CHO supercompensation models for Olympic triathlon competitions. Understanding how triathletes program CHO supercompensation and supplementation can help sport nutritionists suggest better optimized CHO supercompensation and supplementation plans.

The aims of the present study were to investigate triathletes' knowledge about CHO supercompensation and supplementation strategies, how they manage these strategies prior to Olympic triathlon competitions, and whether self-reported CHO supercompensation and supplementation strategies are in line with the best practices supported in the literature (8).

## Material and Methods

The target sample were triathletes who had previously competed in the Olympic triathlon category. Athletes were contacted through disclosure on social networks and invited to answer a survey upon signing an informed consent form. The study was approved by the Research Ethics Committee of Federal University of Alagoas (CAAE number: 43129220.0.0000.5013).

The survey was conducted during the COVID-19 pandemic using an online questionnaire in the Google Forms platform. The questionnaire included multiple-choice and open questions. The first part focused on overall information about participants, such as experience in triathlon (time of practice) and training features (weekly volume and frequency). The second part focused on nutrition and training information referring to the week prior to the last Olympic triathlon competition. In order to do so, participants provided detailed information about CHO supercompensation and supplementation strategies, training volume, gastrointestinal discomforts, use of other performance-oriented supplements, and whether they received professional nutritional support.

The CHO supercompensation strategies were classified as: 1) classic CHO supercompensation model, when CHO intake was low (lower than usual) from the 6th to the 4th day prior to the competition, followed by high CHO (higher than usual) from the 3rd day prior to the competition until the competition day (5); 2) modified CHO supercompensation model, when CHO intake was high from the 3rd day prior to the competition until the competition day, and the athlete did not perform any muscle glycogen depletion phase (7); and 3) updated CHO supercompensation model, when CHO intake was high only 24 h prior to the competition, and the athlete did not perform any muscle glycogen depletion phase (6). The training volume was defined by the number of hours trained by the participants. Tapering was considered if the participant significantly reduced the number of hours (volume) trained in the week of the competition.

The inclusion criteria were: ≥18 years old, had completed an Olympic triathlon event, and able to understand and answer questions in Portuguese. The athletes who performed different types of triathlons or provided incomplete answers were excluded.

### Statistical analysis

Data were tabulated in a Microsoft Excel^®^ spreadsheet. Shapiro-Wilk test was applied to check data normality. Data are reported as means±SD or absolute and relative frequency. The *t*-test for independent samples and chi-squared test were used to check differences between sexes. Analyses were carried out in RStudio software, version 3.6.2 (Austria), and significance was set at P<0.05.

## Results

Eighty-nine triathletes responded to the questionnaire. Seventeen questionnaires were excluded from analysis because they were filled out by triathletes in sprint (n=11), duathlon (n=1), and ironman (n=5) categories. The remaining 72 questionnaires completed by Olympic triathletes were fully analyzed. Respondents were 84.7% male (n=61) and 15.3% female (n=11).

A larger number of female athletes (chi-squared test, P=0.034) were under the advice of certified sports nutrition coaches (90.9%) than male athletes [57.8% (certified sports nutrition coaches=55.7% + medical doctor=1.6%)]. Women were shorter, lighter, and dedicated longer hours to usual training on a weekly basis, mostly routine running training, than men. Women also dedicated longer hours to training, mainly to swimming training, during the week prior to competitions, than men. Athletes reduced their training volume in the seven days prior to competitions; this reduction was applied to all training types by both women and men (Table 1). Swimming training volume in the competition week was maintained at 79.3±25.9% for men and 78.7±24.7% for women, whereas cycling training volume was maintained at 56.9±20.9% and 59.1±22.3%, running training volume was maintained at 67.4±25.4% and 56.9±8.48%, and strength training volume was maintained at 47.1±42.9% and 53.6±42.4%, for men and women, respectively. Overall training volume decreased to 63.7±17.6% among men and to 64.1±15.8% among women in relation to usual training.

**Table 1:** Descriptive features of Olympic triathletes participating in the study.

	Gender		P-value
	Male (n=61)	Female (n=11)	
Age (years)	38.9±9.4	30.2±7.7	0.671
Height (meters)	1.76±0.06	1.60±0.04	<0.001*
Weight (kg)	73.6±12.0	55.8±4.3	<0.001*
Triathlon practice time (years)	4.12±4.70	3.44±3.20	0.647
Usual training			
Swimming (h/week)	3.19±1.73	4.27±1.06	0.050
Cycling (h/week)	5.75±1.90	6.73±1.21	0.105
Running (h/week)	3.54±0.98	4.77±1.29	<0.001*
Strength training (h/week)	1.86±1.22	2.01±0.89	0.680
Total	14.14±4.06	17.79±2.52	0.005*
Training during the week prior to the competition			
Swimming (h/week)	2.36±0.92	3.26±0.96	0.005*
Cycling (h/week)	3.25±1.35	4.02±1.83	0.103
Running (h/week)	2.35±0.87	2.77±1.17	0.165
Strength training (h/week)	1.07±1.09	1.24±1.05	0.620
Total	9.03±2.64	11.29±2.85	0.012*
Training during the week prior to the competition (% of usual training)			
Swimming (%)	79.3±25.9	78.7±24.7	0.964
Cycling (%)	56.9±20.9	59.1±22.3	0.759
Running (%)	67.4±25.4	56.9±8.48	0.167
Strength training (%)	47.1±42.9	53.6±42.4	0.644
Total	63.7±17.6	64.1±15.8	0.946

Data are reported as mean and SD. *P<0.05; independent sample *t*-test.

### Carbohydrate supercompensation strategy

CHO supercompensation prior to competitions was reported by 48.6% (n=35) of participants, whereas the remaining 51.4% (n=37) reported maintaining their regular diet until the competition day. The updated CHO supercompensation model was the most mentioned type (27.8%, n=20) followed by both the modified CHO supercompensation model (18.0%, n=13) and the classical CHO supercompensation model (2.8%, n=2). In addition, only 26 (57.7%) athletes among those who consulted a dietician (n=45) performed CHO supercompensation.

### Carbohydrate supplementation strategy

CHO supplementation during competitions was reported by 86.1% (n=62) of participants, whereas the remaining 13.9% (n=10) reported not using CHO supplementation during these events. The CHO supplementation group consumed a mean total of 58.3±37.6 grams of CHO. The estimated rate of CHO intake was 22.1±14.9 g/h, because participants reported that the race lasted from 109 to 214 min (161±25 min). Only two athletes reported supplementation >60 g/h, whereas most of them consumed less than this amount (n=10 consumed ≥30-60 g/h, n=40 consumed <30 g/h). In addition, 10 athletes did not specify their amount of supplementation. CHO types used by respondents comprised: 1) polysaccharides and fructose (51.0%, n=24); 2) polysaccharides, disaccharides, and fructose (27.6%, n=13); 3) only disaccharides (10.6%, n=5); 4) polysaccharides and disaccharides (6.3%, n=3); and 5) polysaccharides (4.2%, n=2). Fifteen participants (28.83%) did not specify the carbohydrate type they used. In addition, 39 (86.66%) participants among those who consulted a dietician (n=45) performed CHO supplementation.

### Gastrointestinal discomfort

Gastrointestinal discomfort was reported by one participant (1.4%) during the CHO supercompensation phase (updated CHO model), by five participants (6.9%) during CHO supplementation phase, and by four participants (5.5%) during both CHO supercompensation (n=3, in the updated CHO supercompensation and n=1, in the modified CHO supercompensation) and CHO supplementation phases. Two of the 9 participants who reported discomfort after CHO supplementation had it during cycling, whereas seven participants had it during running.

### Performance-oriented supplements

Performance-oriented supplements used prior to competitions comprised caffeine (n=26, 36.1%), beta-alanine (n=24, 33.3%), taurine (n=18, 25.0%), sodium bicarbonate (n=1, 1.3%), and guarana powder (n=1, 1.3%). Performance-oriented supplements used during competitions comprised caffeine (n=20, 27.8%), taurine (n=7, 9.7%), beta-alanine (n=1, 1.3%), creatine (n=1, 1.3%), sodium bicarbonate (n=1, 1.3%), and guarana powder (n=1, 1.3%). Most athletes used more than one supplement before or during competitions.

## Discussion

The main findings in the present study were: 1) a little less than half (48.6%) of triathletes used the CHO supercompensation strategy prior to competitions - the updated CHO supercompensation followed by the modified CHO supercompensation were the most reported strategies; 2) most triathletes adopted the CHO supplementation strategy during competitions at a concentration of about 20 g/h; 3) most triathletes who adopted the CHO supercompensation or supplementation strategy were advised by a certified nutrition specialist, whereas one was advised by a physician; and 4) the number of women under counseling by certified nutrition experts was larger than that of men.

Most participants who performed supercompensation followed the most current strategies, whereas only two participants adopted the classical supercompensation strategy. In addition, 19 certified professionals did not comply with nutritional recommendations and prescribed the supercompensation strategy, whereas 12 participants performed it on their own, without consulting any professional in the field. The CHO consumption strategy is supported by its effects on endurance exercise performance (9). In addition, Olympic triathletes often consume high amounts of CHO before the race (16). Furthermore, this finding was in compliance with previous findings that the CHO supercompensation strategy is used by 55% of middle- and long-distance runners (17) and individual road cyclists (18). However, it is worth pointing out that these modalities are single modality sports, whereas Olympic triathlon is a multidisciplinary sport.

Although supercompensation is expected to improve endurance performance, the literature in this field recommends using the updated CHO supercompensation model (8), since in this strategy it only takes one day for athletes to reach maximum glycogen stores. In addition, the updated strategy avoids the fatigue associated with the depletion period, compared to the classical CHO supercompensation model (6). These protocols have the same purpose, which is improving exercise performance through increasing glycogen storage and physiological pathways underlying this adaptation (19,20). For example, during long-duration tasks, glycogen is preferentially degraded in type I fibers (19). In addition, this depletion occurs preferentially in intra-myofibrillar environments, regions with low glycogen stores compared to inter-myofibrillar and sub-sarcolemmal regions (19). However, CHO ingestion can ensure that endurance athletes increase their glycogen stores primarily in the intra-myofibrillar region, attenuating the fatigue process associated with glycogen depletion. In addition, low glycogen levels are associated with decreased calcium (Ca^2+^) release during exercise (20), which can lead to skeletal muscle fatigue, since, during the skeletal muscle contraction process, Ca^2+^ release from the sarcoplasmic reticulum plays an important role in the occurrence of the actin and myosin coupling cycle (21). Therefore, CHO supercompensation promotes a variety of physiological changes that may improve exercise performance during long-duration efforts.

There is no benefit in increasing the initial level of muscle glycogen for endurance exercises lasting less than 90 min, since significant amounts of glycogen remain in the muscles at the end of these exercises (2). Fatigue usually occurs when muscle glycogen levels fall to critically low levels (25 mmol/kg wet weight). This can occur even at normal blood glucose levels (5 mmol/L), indicating that the absolute demand for glycogen in contracting muscle fibers cannot be fully compensated for by the uptake and oxidation of blood glucose (2). Sherman et al. (7) investigated the effect of carbohydrate supercompensation in a 20.9-km run completed by participants in approximately 83 min, but found no significant difference in trial time between the supercompensation and mixed diet groups. Additionally, participants finished the run with approximately 96 mmol/kg wet weight of muscle glycogen, a value higher than the critical point of fatigue. This suggests that shorter events are not capable of depleting muscle glycogen, whereas in longer events, a high level of muscle glycogen at the beginning can delay fatigue by about 20% and improve performance by about 2 to 3% in endurance events longer than 90 min (2), such as the Olympic triathlon, where elite athletes take about 110 min (14) and amateur athletes about 150 min (15) to complete.

CHO supercompensation implementation time meets the training volume reduction time (i.e. tapering). It has been suggested that tapering should take two weeks, based on exponential reduction in training volume by 41-60% (22), without any change in training intensity or frequency (4,22,23), to help maximize athletes' endurance performance. Moreover, based on the exponential model, training volume should reach 30% or less in the last week before the competition (4), which corresponds to approximately half of what participants in the current study have done (63.7±17.6% and 64.1±15.8% for men and women, respectively). Apparently, the participants did not reduce the training volume to the recommended level in the week before the last competition. In total, 53.48% of triathletes reduced training intensity during the tapering period, although general recommendations advise athletes to reduce training volume and maintain or increase intensity (4,22,23).

Most triathletes had approximately 60 g CHO supplementation during the race. Considering that the race lasted about 160 min, the estimated rate of CHO use was close to 20 g/h. Interestingly, only two athletes reported taking >60 g/h supplementation, whereas most participants took lower doses that were probably not effective for an Olympic triathlon. The current recommendation for CHO supplementation in events lasting 2 or more hours is 90 g/h CHO (8,9), suggesting that the participants of this study had insufficient CHO supplementation for the race. Despite the insufficient CHO level, most participants combined different CHO types, which could provide additional endurance performance if sufficient CHO amount was ingested. Benefits of glucose and fructose intake for endurance performance have been observed in exercise activities lasting 2.5 h or longer; they are even greater within the third hour of exercise (12). CHO from multiple sources may not provide additional performance benefits compared to glucose alone in shorter exercises (10,12), since the recommendation for carbohydrate supplementation is less than 60 g/ h for this type of exercise (9,12).

In the present study, more women than men adopted nutritional strategies based on the guidance of certified nutrition specialists. Although many studies on self-reported nutritional strategies are mentioned in the literature (17,24), there is scarce evidence on sex differences in adopting professional recommendations. However, the higher number of women with a nutrition specialist is associated with a responsible sense of food consumption in a wide range of contexts, for example, family environment (25). In this sense, it is expected that women seek professionals in the sports field, since they tend to have more self-care practices than men (25). However, the women did not adhere closely to nutritional recommendations regarding carbohydrate supercompensation and supplementation strategies, similar to the men.

Some triathletes presented gastrointestinal discomfort during CHO supercompensation and/or supplementation. However, this discomfort prevailed mostly in the running phase of the race. It is believed that gastrointestinal discomfort prevails in the running phase because it is the last part of the Olympic triathlon, being thus more due to the race duration, rather than to the running itself. It has been described that prevalence for gastrointestinal distress is augmented with increasing exercise duration (26), possibly due to increasing dehydration and decreased blood supply to the gastrointestinal tract (27). However, a study that assessed athletes who engaged in cycling after running found more gastrointestinal symptoms in the running activity than in the cycling one (28). This finding suggests that running induces more gastrointestinal discomfort, which may be linked to exacerbated bowel movements during this activity (29). Other factors, such as weather conditions and athletes' training level, can also influence gastrointestinal discomfort rates (30).

The carbohydrate supplementation strategy can also influence gastrointestinal discomfort. Glucose is absorbed from the intestinal lumen via the sodium-dependent transporter SGLT1 (11,31), which can become saturated at ingestion rates greater than 1-1.2 g/min or ∼60 g/h (13). In contrast, fructose is absorbed via the GLUT5 transporter (11,31). The combination of glucose and fructose results in a higher rate of exogenous carbohydrate oxidation and fewer gastrointestinal problems compared with ingestion of glucose alone (32). This is due to the reduced competition for intestinal transport, as glucose and fructose utilize distinct transport mechanisms (SGLT1 and GLUT5, respectively). Therefore, for training or competition benefits from a CHO intake greater than 60 g/h (approximate value of SGLT1 saturation), co-ingestion of glucose and fructose is recommended, as they do not compete for intestinal transport. Intestinal tract training, also known as “gut training”, can help improve CHO absorption during exercise and mitigate gastrointestinal symptoms to reduce the risk of gastrointestinal discomfort (33). Thus, endurance nutritional strategies should be used during the training period to allow athletes to adapt to higher carbohydrate volume intake. In doing so, the regular intake of carbohydrate-rich diets significantly increases the SGLT1 content in the intestinal lumen, as does training right after a meal and supplementing with a large carbohydrate amount during training. This process simulates a nutritional plan aimed at competition, and it enables higher carbohydrate absorption and oxidation levels during exercise, reducing the athletes' gastrointestinal discomfort (33).

Some athletes turned to supplements to improve both their race time and performance. Caffeine, beta-alanine, and taurine were the most commonly used supplements. The use of caffeine among the athletes was in accordance with previous studies showing that 89% of ironman athletes (34) and 25% of triathletes, ∼20% of runners, and 24% of cyclists (35) ingested caffeine before the task. Caffeine intake is associated with its mechanisms of action in endurance sports, such as antagonism of the effects of adenosine (36), which results in increased neuro excitability, reduced perceived exertion (36), and more intense contractions during exercise (36). Furthermore, the popularity of caffeine may be due to evidence showing that only unusually high doses (i.e. >9 mg/kg) of caffeine generate gastrointestinal discomfort responses (37). Furthermore, daily supplementation with 4-6 g of beta-alanine for 2-4 weeks improved individuals' exercising performance - the strongest effects being observed in tests lasting 1 to 4 min (38). However, its ergogenic effect on endurance exercises longer than 25 min remains uncertain (38), suggesting that beta-alanine is not a supplement for triathlon athletes. On the other hand, a meta-analysis of ten studies has shown that acute or chronic taurine intake ranging from 1 to 6 g/d improved individuals' aerobic performance (39). However, divergent results regarding the potential ergogenic effect of taurine on performance do not allow definitive conclusions to be drawn regarding the recommendation of its use. The consumption of beta-alanine and taurine identified in the present study may be associated with the lack of knowledge of athletes about the correct use of these ergogenic aids.

The present study had some limitations, such as memory bias, lack of an interviewer, and possible sampling bias due to disclosure on social media. Furthermore, the amateur athletes did not reduce training volume sufficiently during the week prior to the competition as recommended by the tapering protocol, and even reduced the intensity when general recommendations advise athletes to reduce training volume and maintain or increase intensity. This may not guarantee a similarity of glycogen content prior to the competition. In addition, the athletes performed different competitions, and variations such as humidity and temperature were not considered. In this sense, future studies can overcome these limitations by increasing the accuracy of the questionnaire and focusing on questions that are more relevant than others (40). Furthermore, a focus should be placed on simulating Olympic triathlon and testing self-reported and literature-based CHO supplementation and supercompensation strategies to help understand recommended CHO plans prior to Olympic triathlon competitions. Moreover, studies involving longer duration triathlon categories, such as half-Ironman and Ironman, in which CHO plans may be more important, are also recommended. These tasks result in more prolonged disturbance of the internal environment. Thus, a better and more specific understanding of CHO intake strategies in these other triathlon categories may improve targeted nutritional recommendations.

In conclusion, most of the 72 participants of this study were men and reported adopting tapering before competitions and using CHO supplementation during the race. However, they did not follow any supercompensation strategy prior to competitions, in contrast to the literature. Yet, those who reported using CHO supplementation during the triathlon did not follow the recommended amount-per-hour. Thus, the small number of triathletes performing supercompensation and the insufficient amount of supplemented carbohydrate suggest that triathletes are either unaware of the nutritional recommendations or choose not follow them.
